# The management and outcome of hyponatraemia following transsphenoidal surgery: a retrospective observational study

**DOI:** 10.1007/s00701-022-05134-9

**Published:** 2022-01-25

**Authors:** Ziad Hussein, Ploutarchos Tzoulis, Hani J. Marcus, Joan Grieve, Neil Dorward, Pierre Marc Bouloux, Stephanie E. Baldeweg

**Affiliations:** 1 Department of Endocrinology, University College London Hospital, London, UK; 2 Division of Medicine, University College London, London, UK; 3 Department of Endocrinology, The Whittington Hospital, London, UK; 4 Department of Neurosurgery, National Hospital for Neurology and Neurosurgery, London, UK; 5 Centre for Neuroendocrinology, Royal Free Campus, University College Medical School, University College London, London, UK

**Keywords:** Hyponatraemia, Transsphenoidal surgery, Syndrome of Inappropriate ADH secretion, Pituitary adenoma

## Abstract

**Purpose:**

Hyponatraemia is a common complication following transsphenoidal surgery. However, there is sparse data on its optimal management and impact on clinical outcomes. The aim of this study was to evaluate the management and outcome of hyponatraemia following transsphenoidal surgery.

**Methods:**

A prospectively maintained database was searched over a 4-year period between January 2016 and December 2019, to identify all patients undergoing transsphenoidal surgery. A retrospective case-note review was performed to extract data on hyponatraemia management and outcome.

**Results:**

Hyponatraemia occurred in 162 patients (162/670; 24.2%) with a median age of 56 years. Female gender and younger age were associated with hyponatraemia, with mean nadir sodium being 128.6 mmol/L on postoperative day 7. Hyponatraemic patients had longer hospital stay than normonatraemic group with nadir sodium being inversely associated with length of stay (*p* < 0.001). In patients with serum sodium ≤ 132 mmol/L, syndrome of inappropriate antidiuretic hormone secretion (SIADH) was the commonest cause (80/111; 72%). Among 76 patients treated with fluid restriction as a monotherapy, 25 patients (25/76; 32.9%) did not achieve a rise in sodium after 3 days of treatment. Readmission with hyponatraemia occurred in 11 cases (11/162; 6.8%) at a median interval of 9 days after operation.

**Conclusion:**

Hyponatraemia is a relatively common occurrence following transsphenoidal surgery, is associated with longer hospital stay and risk of readmission and the effectiveness of fluid restriction is limited. These findings highlight the need for further studies to better identify and treat high-risk patients, including the use of arginine vasopressin receptor antagonists.

**Supplementary Information:**

The online version contains supplementary material available at 10.1007/s00701-022-05134-9.

## Introduction

Postoperative hyponatraemia, defined as serum sodium value less than 135 mmol/L within 30 days of surgery, is a frequent complication following transsphenoidal surgery for pituitary adenoma, with a reported incidence of 16–23%[1, 2, 19, 33, 36]. The most common aetiology is syndrome of inappropriate antidiuretic hormone secretion (SIADH) as a consequence of surgical manipulation of the neurohypophysis and hypothalamus[2, 14, 15]. Other causes include cerebral salt wasting syndrome (CSWS), hypocortisolism due to adrenocorticotropic hormone (ACTH) deficiency, severe hypothyroidism, overzealous desmopressin (DDAVP) administration and hypotonic fluid infusion[2, 13]. The optimal therapeutic strategy for postoperative hyponatraemia differs according to its cause. Fluid restriction remains the mainstay of treatment in SIADH, fluid and sodium replenishment are the treatment of choice in CSWS, whereas glucocorticoid and thyroxine replacement are required for adrenal and thyroid deficiency, respectively.

Despite recent initiatives in the USA and the UK[4, 24], hyponatraemia remains the leading cause of unplanned hospital readmissions within 30 days of transsphenoidal surgery for pituitary tumours[3, 4, 6]. To this end, the aim of this study was to evaluate the management and outcome of hyponatraemia following transsphenoidal surgery.

## Methods

### Study design and population

A retrospective case control study design was adopted, and the study was registered and approved by the local Clinical Governance Committee. The Strengthening the Reporting of Observational Studies in Epidemiology (STROBE) Statement was used in the preparation of this section of the manuscript[9].

A prospectively maintained database was searched over a 4-year period between 1st January 2016 and 31st December 2019, to identify all patients undergoing transsphenoidal surgery that were found to have serum sodium less than 135 mmol/L during their hospitalisation. The study was conducted at the National Hospital for Neurology and Neurosurgery, which performs the highest volume of pituitary operations in the UK. Operations were performed by three experienced neurosurgeons using either an operating microscope (JG) or endoscope (HJM and NLD).

### Data collection

Demographic, clinical, laboratory and radiological data were obtained from each patient’s medical records. Pituitary adenomas were classified according to their size and endocrine activity. The pre-operative adenoma size was determined using magnetic resonance imaging (MRI), with pituitary microadenoma being defined as an adenoma with a diameter less than 1 cm and pituitary macroadenoma as an adenoma with a diameter equal or more than 1 cm. The diagnosis of functioning and non-functioning pituitary adenomas was based on standard endocrine assessment and histological analysis of the resected tumours. Histological diagnosis of other sellar and parasellar lesions was also recorded.

All patients underwent measurement of anterior pituitary hormones prior to surgery and on the second post-operative day. Patients noted to have secondary adrenal insufficiency and/or hypothyroidism were treated with replacement therapy. Hypocortisolism was defined as early morning serum cortisol less than 350 nmol/L, and secondary hypothyroidism was defined as free T4 level less than 12 pmol/L (reference range 12–22 pmol/L).

Serum sodium was recorded at baseline preoperatively and daily during the first week postoperatively. Hyponatraemia in this study was classified into mild (serum sodium level between 130 and 134 mmol/L), moderate (serum sodium level between 125 and 129 mmol/L) and severe hyponatraemia (serum sodium level less than 125 mmol/L)[35]. Nadir sodium is the lowest sodium level measured at any point within 30 days following transsphenoidal surgery. In our institution, serum sodium of 132 mmol/L is considered clinically meaningful and prompts management. Therefore, data on the management and outcome of hyponatraemia were extracted for those who developed serum sodium ≤ 132 mmol/L following surgery. Renal function, serum osmolality, urine osmolality and sodium, serum cortisol and thyroid function were also recorded. The diagnosis of SIADH was made in those who met the diagnostic criteria of clinical euvolaemia, measured serum osmolality < 275 mOsm/kg, urine osmolality > 100 mOsm/kg and urine sodium > 30 mmol/L, as well as normal adrenocortical and thyroid function, without recent administration of diuretics[35]. Data on treatment and serum sodium level post treatment initiation were recorded. Data on short-term outcomes, including intensive care unit admission and length of hospital stay, as well as on readmission rate were collected.

### Statistical analysis

Basic data were evaluated using descriptive statistics. Mean and standard deviation (SD) were used to describe continuous variables. Median and interquartile range (IQR) were used to describe data not normally distributed. Chi square test was used to compare categorical variables including the trend in postoperative sodium level. Linear regression analysis was used to evaluate the relationship of nadir sodium with patient’s age, gender and length of stay. Statistical significance was defined as *p* value < 0.05. Statistical analysis was performed using GRAPHPAD PRISM 8 software.

## Results

### Baseline characteristics and risk factors for hyponatraemia in all patients with serum sodium level < 135 mmol/L

Among 670 patients who underwent transsphenoidal surgery over 4 years, 162 patients (162/670; 24.2%) developed hyponatraemia (serum sodium < 135 mmol/L) postoperatively. Mild hyponatraemia occurred in 90 patients (90/670; 13.4%), 38 patients had moderate hyponatraemia (38/670; 5.7%) and severe hyponatraemia was recorded in 34 patients (34/670; 5.1%).

**Fig. 1:**
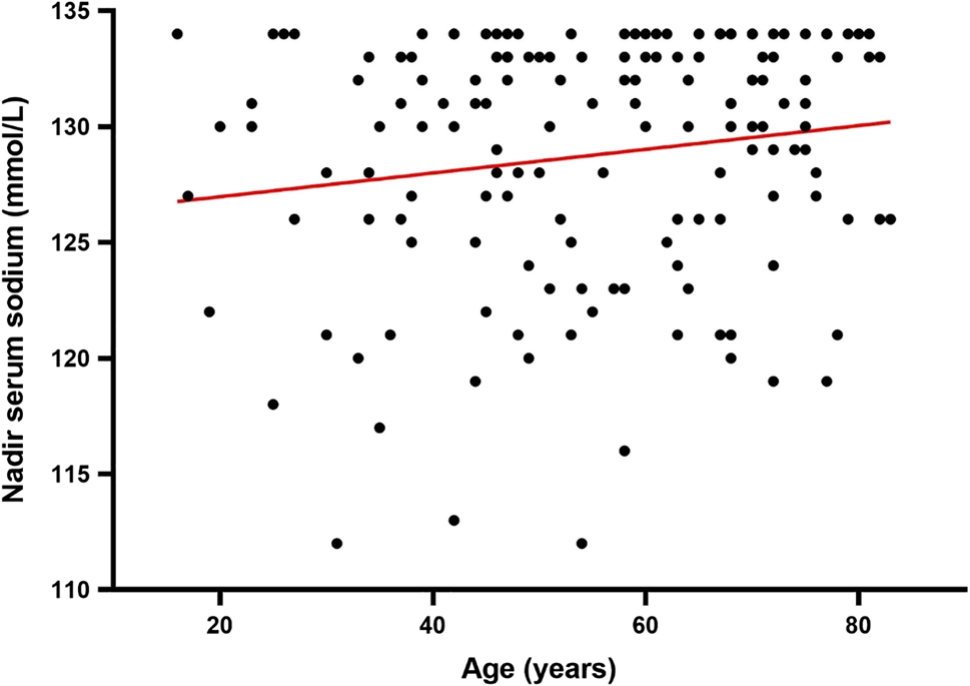
Linear regression analysis of age and nadir serum sodium. X axis represents patients’ age in years, and Y axis represents nadir serum sodium (mmol/L). Individual sodium levels plotted as dark circles

Patients’ characteristics, tumour histology and surgical approach are shown in Table 1. The median age for hyponatraemic patients was 56 years (IQR 44–68). Younger age was associated with hyponatraemia (*p* = 0.043). On average, the value of nadir sodium increased by 0.05 mmol/L for each year increase of age (*F*(1,159) = 4.18, *p* = 0.043, *R*^2^ = 0.03) (Fig. 1).

**Table 1:** The incidence of hyponatraemia among all patients treated with transsphenoidal surgery. Patients’ characteristics, surgical technique and tumour pathology are represented in numbers and percentages

	Hyponatraemia (< 135 mmol/L)	Normonatraemia (135–145 mmol/L)		Total	*p* value
Patient’s gender					
Male	77 (22.9%)		259	336	*p* = 0.4
Female	85 (25.4%)		249	334	
Surgical technique					
Microscopic surgery	107 (25.2%)		316	423	*p* = 0.4
Endoscopic surgery	55 (22.2%)		192	247	
Tumour pathology					
Pituitary adenoma	122 (22.3%)		424	546	
Craniopharyngioma	11 (39.2%)		17	28	
Rathke’s cleft cyst	6 (23.1%)		20	26	
Meningioma	6 (23.1%)		20	26	
Pituitary metastasis	3 (30%)		7	10	
Pituitary inflammation	1 (11.1%)		8	9	
Epidermoid cyst	2 (50%)		2	4	
Pituicytoma	1 (33.3%)		2	3	
Rare pathologies (dermoid cyst, glioneuronal tumour, germinoma, infection, chordoma, cavernoma, glioma, unknown)	10 (55%)		8	18

There was no difference in the incidence of hyponatraemia observed between males and females (*p* = 0.4). However, female gender was significantly associated with lower nadir level (mean sodium = 124.8 mmol/L) than with male gender (mean sodium = 133 mmol/L) (*p* < 0.001).

There was no significant difference in the frequency of hyponatraemia between patients with microadenoma and those with macroadenoma (*p* = 0.7) (Table 2). The frequency of hyponatraemia was similar in patients with pituitary macroadenoma with and without optic nerve (*p* = 0.8). In addition, there was no difference in the frequency of hyponatraemia between patients with functioning and non-functioning adenomas (*p* = 0.5). Although patients with craniopharyngioma developed hyponatraemia at a higher rate than those with pituitary adenoma, this was not statistically significant (39.2% versus 22.3%; *p* = 0.06). With respect to the neurosurgical technique, the occurrence rate of hyponatraemia was similar between microscopic and endoscopic transsphenoidal surgeries (*p* = 0.4).

**Table 2:** The occurrence of hyponatraemia in patients with pituitary adenoma. Patients are represented with numbers and percentages

	Hyponatraemia (< 135 mmol/L)	Normonatraemia (135–145 mmol/L)	Total	*p* value
Pituitary adenoma (total)	122 (22.3%)	424	546	
Adenoma size on radiology				
Microadenoma	15 (20.0%)	60	75	*p* = 0.7
Macroadenoma (total)	107 (22.7%)	364	471	
Macroadenoma without optic nerve compression	28 (21.5%)	102	130	*p* = 0.8
Macroadenoma with optic nerve compression	79 (23.1%)	262	341	
Pituitary adenoma endocrine hyperfunction				
FPA	35 (25.7%)	136	171	*p* = 0.5
NFPA	87 (23.2%)	288	375	
Pituitary adenoma pathology				
Gonadotropin expressing (non-secreting) adenoma	64 (24.3%)	199	263	
Acromegaly	14 (16.6%)	70	84	
Cushing’s disease	15 (22.0%)	53	68	
Null cell	11 (22.4%)	38	49	
Silent corticotroph	4 (12.9%)	27	31	
Functioning prolactinoma	6 (31.5%)	13	19	
Plurihormonal adenoma	4 (21.0%)	15	19	
Non-functioning TSH expressing adenoma	3 (42.8%)	4	7	
Non-functioning GH expressing adenoma	0 (0%)	3	3	
Non-functioning prolactin expressing adenoma	1 (33%)	2	3	

*FPA* functioning pituitary adenoma, *GH* growth hormone, *NFPA* non-functioning pituitary adenoma, *TSH* thyrotropin stimulating hormone

### Incidence, time course and severity of hyponatraemia in all patients with serum sodium < 135 mmol/L

Prior to surgery, ten patients (10/156; 6.4%) had pre-existing hyponatraemia with a level of 132.2 (± 3.5) mmol/L. The median time for serum sodium to decrease below 135 mmol/L was 4 days (IQR 1–6) after tumour resection. The mean (± SD) nadir sodium level for all patients was 128.6 (± 5.2) mmol/L, and the median timepoint to exhibit nadir sodium was postoperative day 7 (IQR 2–8). Nadir sodium according to hyponatraemia severity is shown in Table 3. Nadir sodium varied according to the aetiology. In SIADH, nadir level was 125.1 (± 5) mmol/L with a median time of onset of 8 days (IQR 6–9 days). In patients with adrenal insufficiency, lowest sodium concentration was 130 (± 2.4) mmol/L, occurring after a median time period of 1 day (IQR 1–5). In patients with DDAVP overreplacement, nadir sodium was 127 (± 4.4) mmol/L with a median duration of 7 days (IQR 3–9) postoperatively.

**Table 3:** The timing and impact of hyponatraemia according to severity on regaining normal sodium level and inpatient hospital stay

	Mild hyponatraemia	Moderate hyponatraemia	Severe hyponatraemia
Mean nadir Na (SD)	132.5 (± 1.4)	127 (± 1.2)	120 (± 3.3)
Median time to exhibit nadir Na post TSS (IQR)	3.5 days (1–8)	7 days (2–8)	8 days (7–9)
Median time to achieve normal Na post hyponatraemia therapy (IQR)	2 days (1–3)	4 days (2–6)	6 days (4–9)
Median hospital stay (IQR)	7 days (5–10)	12 days (7–16)	11 days (7–16)

*IQR* interquartile range, *SD* standard deviation, *Na* sodium

**Fig. 2:**
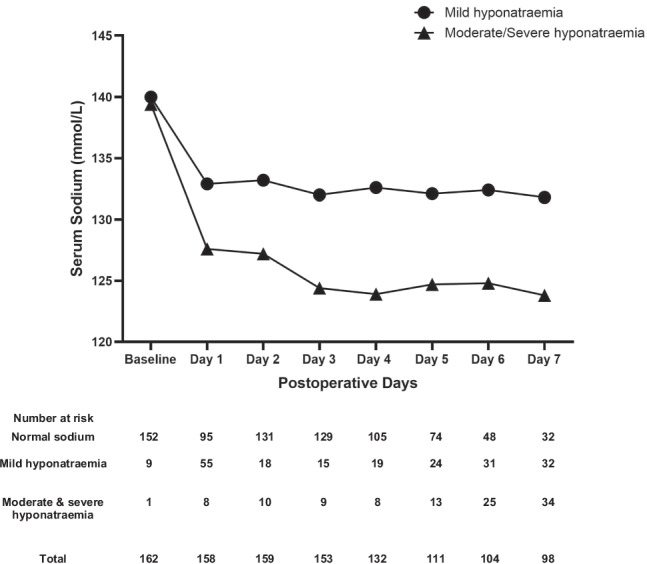
Mean serum sodium during the first 7 days following transsphenoidal surgery according to hyponatraemia severity. Sodium levels in mild hyponatraemia are expressed in black circles, in moderate and severe hyponatraemia in black triangles

We examined the trend of hyponatraemia according to the severity during the first postoperative week. Mild hyponatraemia was more common in the early postoperative period, mostly on day 1 (*p* = 0.0001), while severe hyponatraemia started from day two and evolved in a delayed pattern most commonly on day 7 (*p* = 0.0001) (Supplementary Figure). Mean serum sodium during the first 7 days following transsphenoidal surgery according to hyponatraemia severity is shown in Fig. 2.

### Investigations and aetiology for patients with serum sodium ≤ 132 mmol/L

One hundred and eleven patients had serum sodium levels of 132 mmol/L and below. The commonest cause was SIADH (80/111; 72%), followed by adrenal insufficiency (9/111; 8.1%), overzealous treatment of cranial diabetes insipidus with DDAVP (6/111; 5.4%) and hypotonic hyponatraemia due to hypotonic fluid administration (3/111; 2.7%). There were no documented cases of CSWS. No clear diagnosis was documented in 13 patients (13/111; 11.7%). Table 4 demonstrates biochemical assessment results for patients with SIADH.

**Table 4:** The diagnostic work-up of hyponatraemia for patients with syndrome of inappropriate antidiuretic hormone secretion. Results are expressed in mean levels and standard deviation (SD)

	Mean level (± SD)
Nadir serum sodium (135–145 mmol/L)	125.1 (± 5)
Urea (1.7–8.3 mmol/L)	4.6 (± 1.7)
Serum creatinine (66–112 μmol/L)	63 (± 21)
Serum osmolality (285–295 mOsm/kg)	265 (± 13)
Urinary osmolality (300–900 mOsm/kg)	508 (± 223)
Urinary sodium (mOsm/kg)	80 (± 50)

All patients had early morning cortisol checked 48 h postoperatively. In patients with a new onset of secondary adrenal insufficiency, mean morning cortisol was 138.7 (± 84) nmol/L. Thyroid status was assessed in all patients pre-operatively and postoperatively with mean free T4 level of 18 (± 5) pmol/L (reference range 12–22 pmol/L). Notably, 11 patients had low free T4 level prior to surgery which was treated appropriately with levothyroxine replacement.

### Treatment of hyponatraemia in patients with serum sodium ≤ 132 mmol/L

Fluid restriction was imposed on 83 patients with serum sodium ≤ 132 mmol/L; this included 80 patients with SIADH, 3 patients with fluid overload.

For patients with SIADH, 72 (72/80; 87.8%) had fluid restriction as a monotherapy. Of those, 60 patients had fluid restriction between 500 and 1000 mL daily, and 12 patients reduced fluid intake to 1500 mL daily.

**Fig. 3:**
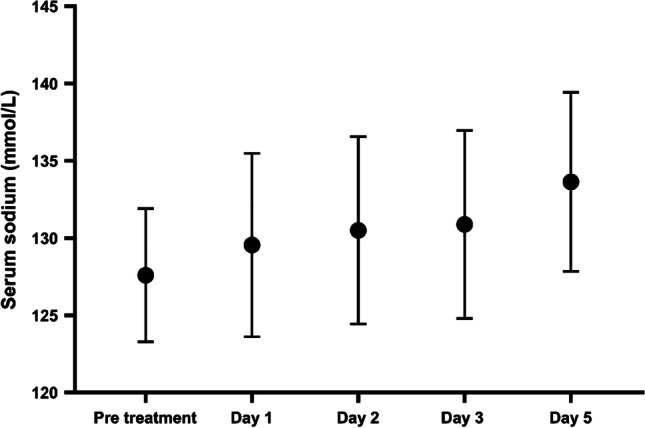
Serum sodium concentration after starting fluid restriction. Sodium levels are expressed as mean and standard deviation. The black circles represent mean sodium levels for those treated with fluid restriction

Fluid restriction as a monotherapy achieved a mean increase in serum sodium of 3.3 mmol/L over a 3-day period. Notably, 23 patients (23/72; 31.9%) did not achieve any increase in sodium levels during the first 3 days of fluid restriction, four patients (4/72; 5.5%) had a mean sodium increase of 1–2 mmol/L, 8 patients (8/72; 11.1%) had 3–4 mmol/L sodium increase and 37 patients (37/72; 51.3%) had sodium increment of ≥ 5 mmol/L during the first 3 days of fluid restriction. The median time to achieve an increase in serum sodium of ≥ 5 mmol/L was 3 days (IQR 2–6 days) and to achieve normal sodium concentration was 4 days (IQR 2–6). Figure 3 demonstrates sodium levels at baseline and after starting fluid restriction in those received fluid restriction only.

Second-line treatment for SIADH was administered in a total of 8 patients; with hypertonic saline 1.8% being used in 5 patients (5/84; 5.9%), hypertonic saline 2.7% in 1 patient (1/84; 1.1%), sodium chloride tablets in 1 patient (1/84; 1.1%) and tolvaptan at a dose of 7.5 mg in one patient (1/84; 1.1%). The patient who was treated with 2.7% hypertonic saline and the second one who received tolvaptan developed overly rapid correction of sodium by more than 10 mmol/L over the first 24 h.

Patients diagnosed with new onset of secondary adrenal insufficiency (number = 9) received glucocorticoid replacement and achieved a mean sodium increase of 8 mmol/L in the first 3 days post therapy. Patients with DDAVP over replacement (number = 6) were treated by dose down titration, leading to a mean sodium increase of 8 mmol/L during the first 3 days of therapy. No patients were treated with urea or demeclocycline.

### Outcome for all patients with hyponatraemia < 135 mmol/L

**Fig. 4:**
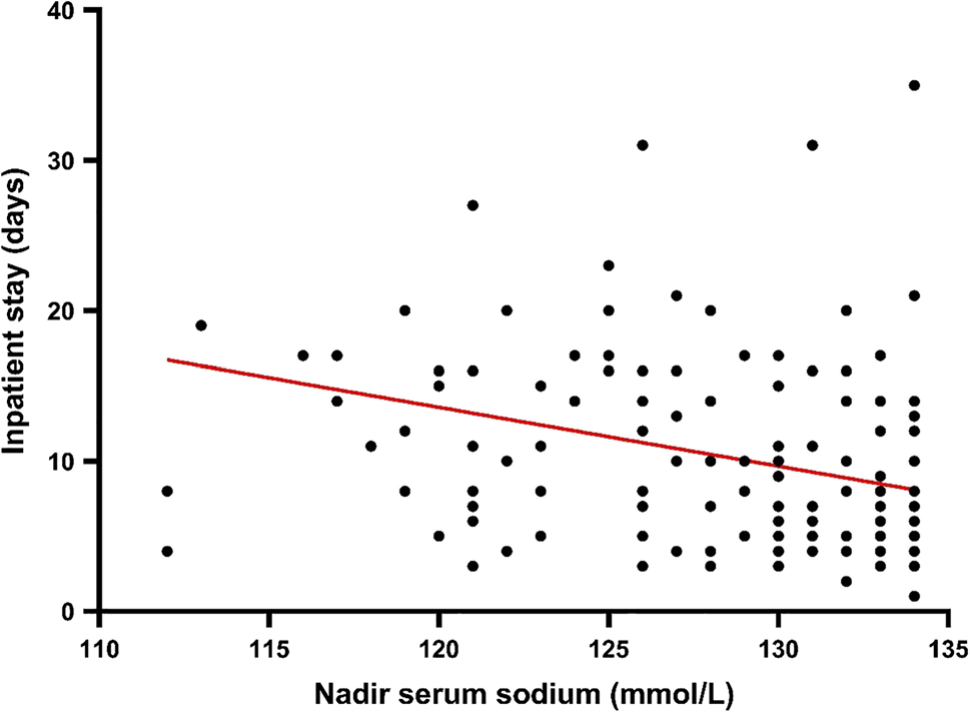
The relationship between mean nadir serum sodium (mmol/L) and in-hospital length of stay in days. In-hospital stay represented in days are expressed as black circles

The mean serum sodium level on discharge for the full cohort was 137.3 (± 4.2) mmol/L. For the full cohort (number = 162), hyponatraemia was corrected in 4 days (IQR 2–6 days), and the length of hospital admission was longer for patients with hyponatraemia (median = 8 days [IQR 5–14]) than the patients who remained normonatraemic throughout hospitalisation (median = 5 days [IQR 4–7]) (*p* < 0.001). Lower nadir sodium was significantly associated with longer inpatient stay. On average, 1 mmol/L reduction in sodium concentration resulted in an increase of inpatient stay by 0.39 days (*F*(1,154) = 14.39, *p* < 0.001, *R*^2^ = 0.09) (Fig. 4).

Seven patients out of 162 (7/162; 4.3%) were admitted to the intensive care unit with a median stay of 3 days (IQR 2–4). One patient had a seizure secondary to severe hyponatraemia. No patients developed permanent neurological morbidities, and there was no associated mortality.

Among 162 patients who developed hyponatraemia, a subset of eleven patients (11/162; 6.7%) had normal sodium levels during initial hospitalisation; however, they were readmitted post discharge with hyponatraemia during the first 30 days of surgery. Those patients were discharged after a median length of initial hospital stay of 4 days (IQR 4–6). Six patients (6/11; 54.5%) were men and five (5/11; 45.5%). The median time of readmission from surgery was 9 days (IQR 7–10). All patients had severe hyponatraemia due to SIADH with a mean sodium level of 120.1 (± 4.4) mmol/L. Among the 11 patients who were readmitted, 8 patients (8/11; 72.7%) were treated with fluid restriction, two (2/11; 18.1%) were treated with both fluid restriction and 1.8% hypertonic saline in the intensive care unit and one (1/11; 9%) patient was treated with a combination of fluid restriction and 2.7% hypertonic saline. The median duration to regain normal sodium levels was 4 days (IQR 3–6), and median duration to hospital discharge was 5.5 days (IQR 3–10).

## Discussion

### Principal findings

This is a real-world study of 670 patients who underwent transsphenoidal surgery in the largest pituitary neurosurgical centre in the UK. We report the following principal findings: (1) the prevalence of post-operative hyponatraemia was 24.2%; (2) female gender and young age were associated with hyponatraemia; (3) tumour size, optic nerve compression, functional status of pituitary adenomas and surgical technique were not predictors for the development of postoperative hyponatraemia; (4) hyponatraemia was mainly due to SIADH, with day 4 being the median time of onset of hyponatraemia and nadir sodium being reported around seven days postoperatively; (5) fluid restriction was used as the treatment strategy in the majority of SIADH patients and was often ineffective in correcting hyponatraemia, leading to prolonged hospitalisation; 6) hyponatraemia was not associated with any long-term neurological sequalae or mortality.

### Comparison with other studies

This study reports a prevalence of post-operative hyponatraemia at 24.2%, in agreement with 16–23% frequency reported in other studies[2, 22, 29, 43, 44]. This series identified two factors associated with postoperative hyponatraemia, female gender and younger age. According to Barber and Zada et al.[2, 45], female gender, but not age, was a risk factor for developing hyponatraemia, while Rajaratnam et al.[31] documented that males had a higher risk of hyponatraemia. The novel finding in this cohort is that the incidence of hyponatraemia increased with younger age. This contrasts with the findings of Hussain et al.[18] and Tomita et al.[36] who reported a link of older age with hyponatraemia, while other reports have not found an association between age and hyponatraemia[2, 19, 45]. In this cohort, tumour size, optic nerve compression, functional status of pituitary adenomas and surgical technique (microscopic versus endoscopic) were not shown to increase the risk of developing hyponatraemia in contrast to other studies[16, 21, 24].

In line with other studies, we confirmed SIADH as the leading cause of hyponatraemia following transsphenoidal surgery. The exact pathogenesis of hyponatraemia following pituitary surgery is not fully understood. Disturbance of fluid balance and, subsequently, of serum sodium because of dysfunctional osmoregulation usually occurs several days after surgical resection, resulting in antidiuresis and hypoosmotic hyponatraemia [26, 28, 39]. Dysregulated secretion of arginine vasopressin (AVP) from the damaged posterior pituitary terminals and magnocellular neurones may result in SIADH and subsequent water imbalance [7, 32]. A prospective study of 92 patients who underwent transsphenoidal pituitary surgery by Olson et al.[28] demonstrated aberrant AVP production with impaired water excretion after water load test in all patients with hyponatraemia and in two-thirds of those with normal sodium. Besides impaired aquaresis, other factors, such as low dietary sodium intake, excessive fluid intake and natriuresis, contributed to the development of hyponatraemia. Hyponatraemia after pituitary surgery has a complex pathophysiological basis and is attributed to multiple factors, including extent of surgical manipulation, damage to the neurohypophysis and hypothalamus, the degree of thirst, fluid volume and dietary sodium intake in the postoperative period. CSWS is a rare but a proposed cause of postoperative hyponatraemia following transsphenoidal surgery. This condition is characterised by excessive renal sodium wasting and circulatory volume depletion in the setting of normal renal and hypothalamic–pituitary–adrenal functions[27]. Despite being first described many decades ago by Peters et al.[30], the causative mechanism and optimal management is still debated. Although it can be challenging in clinical practice, it is considered critically vital to distinguish between SIADH and CSWS, as SIADH is routinely managed with fluid restriction, whereas the main therapy for CSWS is fluid and sodium replenishment. Interestingly, we did not report a single case of CSWS. Our data contradicted the finding by Barber et al.[2] that 24% of hyponatraemic patients post-pituitary surgery had CSWS.

Other, less frequent, causes of hyponatraemia are hypocortisolism, severe hypothyroidism, DDAVP overreplacement and hypotonic fluid administration.

Fluid restriction is the standard first line therapy for the management of SIADH but with controversial efficacy. It is known for its limited effectiveness in treating SIADH, irrespective of its aetiology[2, 12, 41]. In clinical practice, the volume of fluid restriction is generally based on physician’s experience and usually tailored to each individual patient. We recommend using urine/plasma electrolyte ratio formula (urine sodium + urine potassium)/(plasma sodium) as a useful predictive formula to guide water restriction [10]; for example for a ratio of < 0.5 we suggest water limitation up to 1 L/day whereas for a ratio between 0.5 and 1 we suggest restricting fluid to less than 500 mL/day. Others have recommended limiting daily fluid intake to 500 mL below 24-h urine output and use urine osmolality of > 500 mOsm/kg water or urine/plasma electrolyte ratio > 1 as a cutoff to employ pharmacological therapy as a first line treatment instead of fluid restriction [23][23]. Some of the well-recognised issues with fluid restriction are poor tolerance, patient adherence and the difficulty of implementing in clinical practice. We consider patient education by a trained health professional paramount to achieve a successful outcome and encourage patients to do self-fluid balance recording as one of the ways to increase treatment awareness and perception. This series showed that one-third of patients did not respond to fluid restriction within the first 3 days of treatment, while responders required a median time period of 4 days to restore normal sodium. These management outcomes are in contrast to other studies by Burke et al. and Winogard et al.[5, 42]*,* who advocate the implementation of 1000-mL fluid restriction following discharge for all patients during the first week following transsphenoidal surgery, irrespective of whether they have hyponatraemia. They reported successful outcomes of higher sodium levels and less hospital readmission in the interventional group than those who had no restriction. We, however, only managed patients with documented hyponatraemia with fluid restriction during inpatient stay; we think this approach provides more robust data about the efficacy of fluid restriction in treating SIADH as applying treatment in the outpatient setting may be liable to missing some data, particularly with respect to daily serum sodium response. Finally, the mean sodium increase of 3.3 mmol/L after 3-day fluid restriction reported in our study is almost identical to the increase documented in the first ever, and only so far, randomised controlled trial of fluid restriction[11].

Some authors consider commencing tolvaptan if there is no response or suboptimal increase in serum sodium after 24–48 h of fluid restriction[17, 23, 37]. Tolvaptan is a vasopressin receptor antagonist and has been used for the treatment of euvolemic hyponatremia since 2008 with much higher effectiveness in sodium correction than fluid restriction[23, 34, 41]. However, concerns have been raised about the risk of overly rapid sodium correction and subsequent deleterious outcome[25, 38, 40, 41]. A recent study by Kleindienst et al.[22], the only one comparing tolvaptan versus fluid restriction in the treatment of SIADH following pituitary surgery, reported that tolvaptan at a small dose (7.5 mg) was more effective than fluid restriction in the treatment of SIADH but resulted in overly rapid correction of serum sodium in a significant percentage of cases, without shortening the duration of hospitalisation. We recommend using tolvaptan under close guidance of Endocrinologists and Nephrologists with regular monitoring of sodium concentration every 4–6 h following its initiation. If serum sodium incremented by more than 6 mmol/L at 6 h or 10 mmol/L in 24 h, then hypotonic 5% dextrose solution should be administered at a volume matching the urine output to halt further sodium overcorrection[37]. Hypertonic saline 3% is another treatment modality for hypotonic hyponatraemia but is limited to patients with adverse neurological symptoms such as confusion, seizures and dropping Glasgow Coma Scale secondary to hyponatraemia[35][35]. We recommend intravenous infusion of 150 mL 3% solution over 20 min. Treatment can be repeated twice to achieve 5 mmol/L increment in sodium concentration with close monitoring required ideally in the intensive care unit. Other treatment modalities, such as urea, demeclocycline and lithium, are not recommended due to the lack of clear evidence, side-effect profile and limited efficacy[35].

We demonstrated a significant association between hyponatraemia and duration of hospital stay with an increase of 4 days in the duration of hospital stay of hyponatraemic patients compared to those with normal sodium. In addition, the severity of biochemical hyponatraemia was related to the length of hospital stay. Tomita et al.[36] also reported longer hospital stay in those who underwent endoscopic surgery for pituitary adenoma only. We reported this outcome for all sellar and parasellar pathologies.

Unplanned hospital admissions following transsphenoidal surgery are associated with significant clinical and financial implications[20]. Hyponatraemia has been reported as the commonest cause of unplanned readmission following transsphenoidal surgery for pituitary tumours[3, 44]. There is sparse data about the aetiology and predictive factors for rehospitalisation with hyponatraemia[3]. A potential strategy to decrease readmissions due to hyponatraemia would include routine assessment of serum sodium levels in all patients 5–7 days after operation, allowing early identification of hyponatraemia and prompt initiation of fluid restriction on outpatient basis[24]. An alternative pathway to lower readmission rate is to limit fluid intake in all patients post discharge [8, 42]. However, to date there is no general consensus in respect to the best strategy to identify patients at risk and manage delayed hyponatraemia post discharge. This highlights the need to educate patients about the risk of developing delayed hyponatraemia following transsphenoidal surgery and increase patient’s awareness of the clinical symptoms caused by severe hyponatraemia in this setting as well as the importance of measuring serum sodium on days 7–9 after surgery.

### Strengths and weaknesses

The main strength of this study is that it assessed the incidence, management and clinical outcome of hyponatraemia following transsphenoidal surgery in a large cohort of patients. In addition, we reported a detailed impact of different degrees of hyponatraemia on several patient specific clinical outcomes as well as the effectiveness of treatment in those patients which many other studies did not analyse.

This study was based on a retrospective review of medical records and clinical practice, making it subject to limitations of retrospective reports, such as selection bias and incomplete data. Another limitation is the lack of data regarding patient comorbidities and postoperative complications which can be an important confounder contributing to extended in-hospital stay. We observed a high rate of hyponatraemia among patients with rare sellar and parasellar tumours; however, the number of these patients was too small to conclude an association between such tumours and hyponatraemia.

## Conclusions

This study has shown that hyponatraemia, a common complication post transsphenoidal surgery, is associated with prolonged hospital admission and is a common cause of readmission. The limited effectiveness of the current treatment of SIADH highlights the need for prospective studies, evaluating the effectiveness and safety of other therapies for SIADH in this context. Apart from determining the success rate in timely correction of hyponatraemia, these studies should explore the impact of other strategies on outcomes, such as patient’s symptomatology, length of hospital stay and readmission rate. In particular, the potential role of tolvaptan, a V2-specific arginine vasopressin receptor antagonist which is the only medication approved by the regulatory authorities in Europe and the USA for the treatment of SIADH, warrants further exploration in these patients.

## Supplementary Information

Below is the link to the electronic supplementary material.

Supplementary file1 (DOCX 65 KB)

## Data Availability

The data that support the findings of this study are openly available at https://figshare.com/s/455d5b30d05ab9fd520d.
