# Supplementary material for: The management and outcome of hyponatraemia following transsphenoidal surgery: a retrospective observational study
Source: Acta Neurochir (Wien). 2022 Jan 25;164(4):1135–44. doi: 10.1007/s00701-022-05134-9 (PMC8967808; doi:10.1007/s00701-022-05134-9)

**Supplementary Figure.** The distribution of hyponatraemia according to severity during the first 7 days following transsphenoidal surgery. The light grey bars represent mild hyponatraemia, black bars represent moderate hyponatraemia and dark grey bars for severe hyponatraemia.


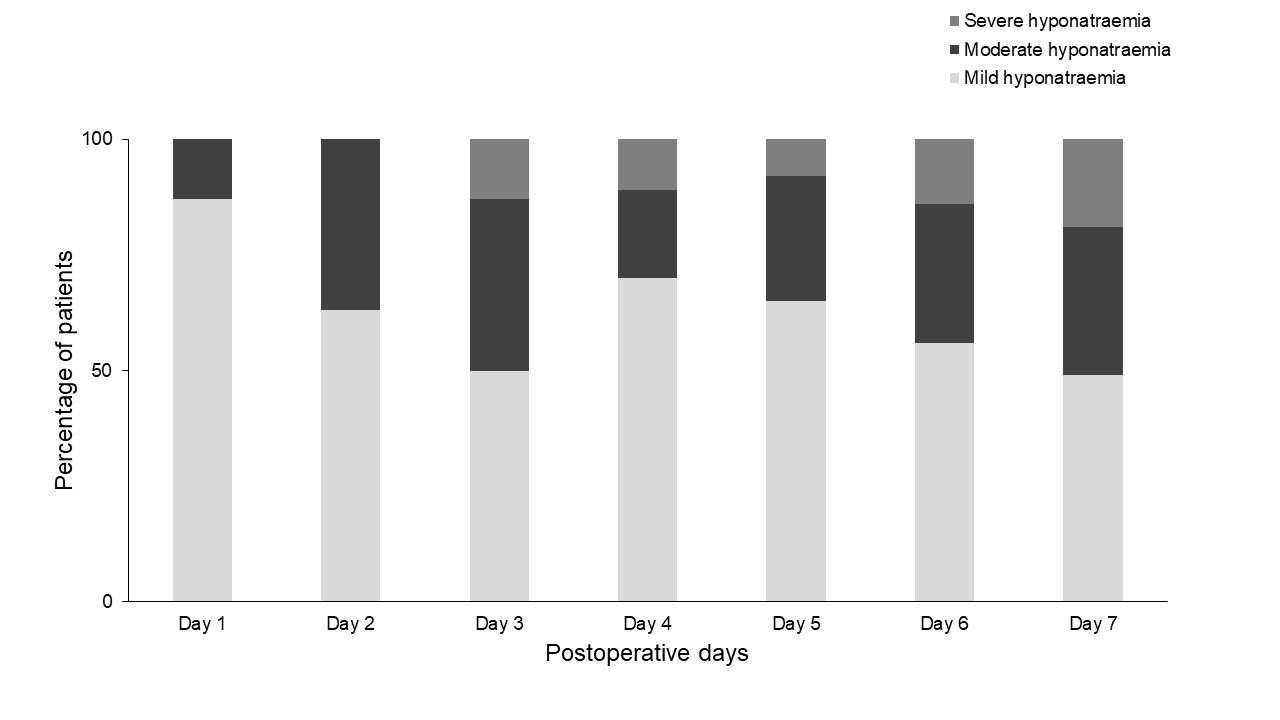

Supplement: Supplementary file 1 — Supplementary file1 (DOCX 65 KB) [file 701_2022_5134_MOESM1_ESM.docx]
